# The VICI-trial: high frequency oscillation versus conventional mechanical ventilation in newborns with congenital diaphragmatic hernia: an international multicentre randomized controlled trial

**DOI:** 10.1186/1471-2431-11-98

**Published:** 2011-11-02

**Authors:** Lieke van den Hout, Dick Tibboel, Sanne Vijfhuize, Harma te Beest, Wim Hop, Irwin Reiss

**Affiliations:** 1 Intensive care and Department of Pediatric Surgery, Erasmus MC - Sophia, Dr. Molewaterplein 50, 3015 GE, Rotterdam, the Netherlands; 2 Department of biostatistics, ErasmusMC, Dr. Molewaterplein 50, 3015 GE, Rotterdam, the Netherlands

## Abstract

**Background:**

Congenital diaphragmatic hernia (CDH) is a severe congenital anomaly of the diaphragm resulting in pulmonary hypoplasia and pulmonary hypertension. It is associated with a high risk of mortality and pulmonary morbidity. Previous retrospective studies have reported high frequency oscillatory ventilation (HFO) to reduce pulmonary morbidity in infants with CDH, while others indicated HFO to be associated with worse outcome. We therefore aimed to develop a randomized controlled trial to compare initial ventilatory treatment with high-frequency oscillation and conventional ventilation in infants with CDH.

**Methods/design:**

This trial is designed as a multicentre trial in which 400 infants (200 in each arm) will be included. Primary outcome measures are BPD, described as oxygen dependency by day 28 according to the definition of Jobe and Bancalari, and/or mortality by day 28. All liveborn infants with CDH born at a gestational age of over 34 weeks and no other severe congenital anomalies are eligible for inclusion. Parental informed consent is asked antenatally and the allocated ventilation mode starts within two hours after birth. Laboratory samples of blood, urine and tracheal aspirate are taken at the first day of life, day 3, day 7, day 14 and day 28 to evaluate laboratory markers for ventilator-induced lung injury and pulmonary hypertension.

**Discussion:**

To date, randomized clinical trials are lacking in the field of CDH. The VICI-trial, as the first randomized clinical trial in the field of CDH, may provide further insight in ventilation strategies in CDH patient. This may hopefully prevent mortality and morbidity.

**Trial registration:**

Netherlands Trial Register (NTR): NTR1310

## Background

Congenital diaphragmatic hernia (CDH) is a severe congenital anomaly of the diaphragm, which occurs in approximately one in 3000 live births [1]. In CDH, the diaphragmatic defect allows abdominal organs to herniate into the chest cavity. As a consequence, underdevelopment of the lungs and abnormal pulmonary vasculature growth may occur, resulting in pulmonary hypoplasia and pulmonary hypertension [2,3].

Children with CDH mostly present with immediate cardiorespiratory distress during the first hours of life. The initial therapy for these children is mechanical ventilation and cardiorespiratory stabilization in support of optimal management of the pulmonary hypoplasia and pulmonary hypertension [4]. Thereafter, surgical repair of the diaphragmatic defect is indicated [4]. Although many advances in treatment for CDH patients have been made throughout the years, CDH remains a life-threatening condition with a reported mortality of 20-70%, depending on case selection [5].

Survivors of CDH are at risk of developing secondary morbidity, such as gastro-intestinal, neurodevelopmental and pulmonary sequelae. Throughout infancy and childhood, a broad spectrum of pulmonary morbidity may occur, such as bronchopulmonary dysplasia (BPD), persistent pulmonary hypertension, recurrent respiratory tract infections and asthmatic symptoms [6]. BPD is characterized by disturbances of the normal alveolarization. Ventilator-induced lung injury, oxygen toxicity, pulmonary inflammatory responses, and severe pulmonary hypertension predispose infants with CDH to develop BPD [7,8]. A recent report of the CDH Registry reported the prevalence of BPD to be 41% in infants with CDH [9]. About half of the CDH survivors with BPD are reported to have moderate to severe BPD [10]. Moreover, lung function anomalies are often present in these patients [11-14].

Optimizing ventilation strategies in patients with CDH may help to prevent chronic respiratory disease. However, evidence-based standardized treatment protocols based are lacking in the field of CDH. Consequently, ventilation strategies may differ between centres and ventilatory support is often based upon expert opinion [7]. To date, conventional ventilation is the most widely used initial ventilation mode in newborns with CDH, while in many institutions high frequency oscillatory ventilation (HFO) is used as rescue therapy. In some centres, however, HFO is used as the initial ventilation mode.

HFO achieves adequate gas exchange by an oscillatory pump, which combines very high respiratory rates and low tidal volumes. HFO may improve gas exchange, promote uniform lung inflation, reduce barotrauma, and decrease the presence of inflammatory mediators [7,15-18]. HFO has been used in preterm infants with respiratory distress syndrome, either as an elective ventilation strategy or as a rescue therapy [16]. A Cochrane review, on the use of elective HFO compared to CMV in preterm infants, found a borderline significant reduction in the rate of chronic lung disease with the use of HFO [16]. A second Cochrane review described the use of HFO as a rescue therapy in term and near term infants with severe pulmonary dysfunction. Only one trial compared these two ventilation strategies prospectively, resulting in no significant difference in outcome, need for extracorporeal membrane oxygenation (ECMO), or complications [15,19].

In infants with CDH, retrospective and observational studies reported improved survival and a lower incidence of chronic lung disease with elective use of HFO [20-25]. HFO was reported to provide better oxygenation and higher MAP without increasing the incidence of barotraumas [20,21]. In one study, the use of HFO avoided hyperventilation as well as the need for ECMO [17]. Moreover, inhaled nitric oxide was documented to result in better gas exchange following recruitment of the lungs by HFO [26]. On the contrary, HFO may cause lung hyperinflation in some patients, which may induce higher alveolar and mean airway pressures [21]. This may result in an increased risk of pulmonary barotrauma and hemodynamic instability [21]. A recent study by the CDH Registry reported HFO as initial ventilation mode to be associated with an increased rate of mortality and BPD [9]. In this study, it is speculated that infants who received initially HFO were more severely ill from the start [9].

Conclusively, chronic pulmonary morbidity and high mortality rates are major problems in infants with CDH. Several retrospective and observational studies have reported that mechanical ventilation strategies may have an impact on outcome and pulmonary morbidity in CDH patients. Therefore, we aimed to develop a randomized controlled trial to compare initial ventilatory treatment with HFO and conventional ventilation in infants with CDH. The acronym VICI is deducted from the words: HFO versus conventional **V**entilation in **I**nfants with **C**ongenital diaphragmatic hernia: an **I**nternational randomised clinical trial. In Latin vici means 'I have conquered' which refers to our patients with CDH which are very vulnerable and ill and sometimes have to struggle to survive.

The primary objective of this trial is to determine if there is a difference in the incidence of BPD and/or death within the first 28 days of life between newborns with congenital diaphragmatic hernia treated with high frequency oscillatory ventilation (HFO) and those treated with conventional mechanical ventilation (CMV) as initial ventilation mode. Secondarily, we also aim to compare the severity of BPD, ventilator-induced lung injury and pulmonary hypertension by using clinical and laboratory parameters.

## Methods/Design

This study is designed as a prospective, randomized controlled multicentre trial. All participating centres are member of the CDH-EURO Consortium. This is a collaboration between European tertiary centres, who have an expertise in the field of CDH. The CDH-EURO consortium was started in 2006 and aims at cooperation between centres, enhancement of research in the field of CDH and development of standardized evidence-based treatment protocols.

### Outcome measures

The primary outcome measure is BPD and/or death within the first 28 days of life. Secondary outcome parameters are overall mortality, severity of BPD, number of days on the ventilator, number of treatment failures, ventilation-induced lung injury and pulmonary hypertension according to clinical and laboratory parameters and need for ECMO (only for ECMO centres). BPD is defined as oxygen dependency at day 28, according to the definition of Jobe and Bancalari [27]. The severity of BPD is also defined according to this definition (see table 1).

**Table 1:** definition of BPD according to Jobe and Bancalari

Gestational age	< 32 weeks	> 32 weeks
**Time point assessment**	36 wks PMA* or DC** to home, whichever comes first	> 28 days but < 56 days of life or discharge to home, whichever comes first
**Treatment with oxygen > 21% for at least 28 days PLUS**		
**Mild BPD**	On room air at 36 wks PMA or at DC, whichever comes first	On room air at day 56 postnatal age or DC, whichever comes first
**Moderate BPD**	< 30% O2 at 36 wks PMA or DC, whichever comes first	< 30% O2 at 56 d of life or DC whichever comes first
**Severe BPD**	≥ 30% O2 and/or pos. pressure at 36 wks PMA or DC whichever comes first	≥ 30% O2 and/or pos. pressure at 56 d of life or DC whichever comes first

*PMA = post-menstrual age

**DC = discharge

### Inclusion and exclusion criteria

The study population consists of all infants antenatally diagnosed with CDH born at one of the participating centres.

Exclusion criteria are:

• Birth before a gestational age of 34 weeks

• Severe chromosomal anomalies, like trisomy 18 or trisomy 13, which may imply a decision to stop or not to start life-saving medical treatment

• Severe cardiac anomalies, expected to need corrective surgery in the first 60 days of life (such as transposition of the great arteries, truncus arteriosus or double outlet right ventricle)

• Renal anomalies associated with oligohydramnios

• Severe orthopaedic and skeletal deformities, which are likely to influence thoracic, and/or lung development (such as chest wall deformities and spine anomalies)

• Severe anomalies of the central nervous system

### Statistical analysis

We estimated that a total of 400 newborns can be included within a period of three years. In a previous study of the CDH study group, the incidence of BPD and/or death within the first 28 days of life is reported to be about 50% [9]. Assuming a difference of 15% in BPD and/or death within the first 28 days between both treatment groups, 186 patients are required per arm for a power of 80% at two-sided alpha is 0.05. To allow for some non-evaluable patients and dropouts, 200 patients per group will be included. To achieve equal distribution of the two ventilation modes among the participants, block randomization stratified per centre will be carried out. The data-analysis will be carried out at the Erasmus MC, Rotterdam. An intention-to-treat analysis will be performed. A p-value (two-sided) < 0.05 will be considered significant in all analyses.

The primary endpoint will be evaluated using multiple logistic regression analysis taking account of: centre, lung-to-head ratio, position of the liver (intra-abdominal or intra-thoracic) and the side of the defect (left, right or central). A subgroup analysis will be carried out in operated infants to evaluate the defect size, according to the CDH study group defect size scale (http://www.cdhsg.net).

The secondary endpoints will be evaluated by using the following statistical tests. Overall mortality in the first year of life will be analysed by Kaplan-Meier curves and Log rank tests. The chi-square test will be used to analyse number of treatment failures, presence of pulmonary hypertension according to echocardiographic parameters, requirement of medication at discharge and during the first year of life, and need for ECMO therapy (in ECMO centres). The Mann-Whitney test is used to evaluate the severity of chronic lung disease, number of days on the ventilator and the fraction of days with required medical treatment for pulmonary hypertension during the hospital admission. In the evaluation of the severity of chronic lung disease and the number of days on the ventilator, deaths are counted as worst outcome according to the intention-to-treat principle. Repeated measures ANOVA is used to evaluate levels of laboratory markers for ventilator-induced lung injury or pulmonary hypertension and pulmonary function at the age of six and twelve months. This analysis allows for missing data at different time points and is considered as the optimal way for evaluation of longitudinal data.

All the above-mentioned analyses will allow for centre by stratification.

### Study procedures

Parental informed consent will be obtained antenatally to enhance quick randomization. Following antenatal diagnosis, the parents are to be counselled and will receive information about the study, including a patient information letter and an informed consent form. If both parents decide to participate in the study, as soon as possible after birth central randomization will take place by the treating physician using a website. This way concealed allocation is guaranteed. Within two hours after delivery, the allocated ventilatory treatment has to be started.

**Figure 1:**
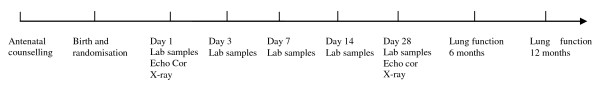
**Overview study procedures**.

The patient receives the allocated ventilation treatment during the entire admission period on the intensive care unit. During surgery, the child preferably remains on the allocated ventilation type, provided the treating surgeon agrees. Should the surgeon opt for the other ventilation mode, the patient is switched to the allocated ventilation treatment again after surgery. If the patient has to be re-intubated within the first 60 days after the start of the allocated treatment, the patient receives the allocated treatment again. Patients are observed up to day 60 after birth or until discharge whichever comes first. In Figure 1, an overview of the study procedures is given.

### Standardized treatment and ventilation settings

All infants participating in the VICI-trial are treated according to the same standardized treatment protocol which has been published recently [4]. This protocol of standard practice was decided upon available evidence and consensus between the participating centres.

After birth, all infants are immediately intubated. In general, our goals are to achieve preductal saturations between 85 and 95%, postductal saturation levels above 70% and arterial CO_2 _levels between 45-60 mm Hg (permissive hypercapnia). Conventional mechanical ventilation is provided by a neonatal ventilator capable of positive pressure ventilation or triggered modes (Babylog ventilator, Dräger Medical, Germany). High frequency oscillatory ventilation (HFO) is provided by a high frequency oscillatory ventilator (Sensormedics 3100A/B HFO Ventilator, Viasys Healthcare, USA).

If the allocated treatment is conventional ventilation, the following settings are used: a peak inspiratory pressure (PIP) of 20-25 cm H_2_O, a positive end-expiratory pressure (PEEP) of 2-5 cm H_2_O and a frequency of 40-60 per minute. In case of HFO, the settings are: a mean airway pressure (MAP) of 13 to 17 cm H_2_O, a frequency of 10 Hz, an amplitude (Δp, cm H_2_O) of 30 to 50 obtaining chest vibrations, and an inspiration/expiration rate (I:E) of 1:1.

According to our standardized treatment protocol, a chest radiograph is made as soon as possible after birth to assess the initial condition and is repeated guided on the patient's condition. In case of HFOV, a chest radiograph is also made after this ventilation mode is started to confirm that the lungs are not overinflated, as defined by a contralateral lung expansion of over 8 ribs. Also, echocardiography is performed within the first 24 hours after birth to assess pulmonary arterial diameter and right heart function. In all patients, a chest radiograph and echocardiography are repeated at day 28.

Weaning from conventional ventilation is done by means of decreasing PIP. Frequency and/or the PIP-PEEP were modified to achieve PaCO_2 _levels above 45 mmHg. In case of HFOV, Δp was decreased if the paCO_2 _was below 45 mmHg and FiO_2 _was decreased if preductal saturation levels were above 95%.

In case of hypovolemia and/or cardiogenic shock, saline fluid therapy is started (10 to 20 ml/kg of NaCl 0.9% up to three times during the first one to two hours), which is followed by inotropic therapy if necessary. At an oxygenation index of 20 or higher and/or a pre- and postductal saturation difference of 10% or more, 10-20 ppm iNO is given for at least one hour. Intravenous prostacyclin or prostaglandin E1 may be considered in case of no response to iNO. Sildenafil may be considered in the chronic phase of pulmonary hypertension.

Surgery is performed if the patient is physiologically stabile according to the criteria described in our protocol [4]. No routine paralysis is used.

### Treatment failure

In case of one or more failure criteria, the allocated ventilatory treatment may be switched. Also, an ECMO procedure may be started in centres where ECMO is available. After the ECMO-procedure has ended, the patient preferably receives the initial allocated ventilation mode. Because an intention-to-treat analysis will be performed, data from these patients are stored and analysed the same way as data from patients in whom no switching of ventilation mode or ECMO procedure took place.

The following failure criteria are applicable to the study patients:

• Inability to maintain preductal saturations above 85% (± 7 kPa or 52 mmHg) or postductal saturations above 70% (± 5.3 kPa or 40 mmHg)

• Increase in CO_2 _> 65 torr or mmHg (8.5 kPa) despite optimalization of ventilatory management

• PIP > 28 cm H_2_O or MAP > 17 cm H_2_O

• Inadequate oxygen delivery with metabolic acidosis defined as lactate ≥ 5 mmol/l and pH < 7.20

• Hypotension resistant to fluid therapy and adequate inotropic support, resulting in a urine output < 0.5 ml/kg/hour

• Oxygenation index consistently ≥ 40

### Laboratory analyses

During the study, blood, urine and tracheal samples are collected. Separate parental informed consent is asked for taking, analysing and storing these samples.

Blood, urine and tracheal samples are taken within the first 2 hours of life and on days 3, 7, 14 and 28. Blood sampling is only done if a central or peripheral line is present and in combination with routine laboratory measurements. Urine sampling is only done if the patient has a urine catheter. Sampling of tracheal aspirates is only done during routine suctioning. In that way, sampling of laboratory markers are of minimal burden for the patient.

#### Blood samples

Blood markers are determined by immuno-assay analysis and are necessary support evidence of chronic lung disease and to further evaluate its severity [28-32].

The following laboratory markers are measured:

• Markers for inflammation and pulmonary vascular endothelial dysfunction and pulmonary hypertension (intercellular adhesion molecule (ICAM), vascular cell adhesion molecule (VCAM), sP- and sE-selectine, pro-brain natriuretic peptide (Pro-BNP), vascular endothelial growth factor (VEGF), von Willebrand factor, thrombomodulin, factor VIII and Asymmetric Dimethylarginine (ADMA)

• Markers for the nitroxide pathway (nitrate and nitrite)

#### Urine samples

Two urine samples from an 8-hour urine collection are taken to measure desmosine, an elastolytic degradation product of elastin. It gives an indication of the degradation of lung elastic fibres in ventilated neonates. One sample of 20 ml containing 0.2% boric acid and one sample of 10 ml will be stored at -20 degrees Celsius. Desmosine will be measured by mass-spectrometry, a highly sensitive way of measuring this laboratory marker [33,34].

#### Tracheal aspirate

Protein profiling by proteomics are used for identifying specific groups of proteins, which are involved in the pathogenesis of chronic lung disease. During routine tracheal suctioning, flushing with 0.5-1.0 ml saline is performed according to standard practice. The tracheal aspirates are centrifuged for 10 minutes at 3000 rpm and are stored at -80 degrees Celsius [35,36].

### Follow-up

At all participating centres, routine follow-up takes place at the age of 6 months, 12 months, 24 months, 5 years, 8 years, 12 years and 18 years in all patients with CDH, including participants of the VICI-trial. During follow-up visits, routine physical examination and additional testing (e.g. lung function tests or developmental tests) take place. For the purpose of this study, parents complete a patient diary card on a daily basis for one month at the age of six and twelve months. By using this diary card information about whether their child coughed, wheezed and/or needed medication is collected. This gives a quantitative assessment of morbidity.

In a subgroup of patients born at the Erasmus Medical Centre and King's College Hospital, lung function tests are performed at the age of six and twelve months. Only these two centres have the equipment and expertise to measure lung function in children below the age of one. At King's College, FRC (functional residual capacity) and LCI (lung clearance index) are measured by using helium gas dilution. The LCI is the cumulative expired volume required to clear an inert gas from the lungs, divided by the FRC. Also, lung function tests are performed before and after bronchodilation at this centre. Therefore, a bronchodilator is administered by a face mask and a spacer. At the Erasmus Medical Centre, the FRC is determined by plethysmographic measurements and the LCI is defined by SF6 measurements.

Since the methods of lung function measurements differ between the two centres, these measurements will be analysed separately for both centres.

### Safety reporting

An adverse event or complication is defined as any undesirable occurrence in a patient. Mortality is regarded a serious adverse event. Adverse events or complications are reported to the study coordinator within 24 hours. Thereafter, the study coordinator informs the data safety monitoring board, who monitors the incidence of mortality on a continuous base. If at some point a large difference in mortality between the two treatment groups is noticed, the data safety monitoring board may recommend ending the study.

### Data collection

Demographic and neonatal characteristics as well as data on the clinical course and treatment of all patients will be collected in a central database in Rotterdam. Corporeal material collected during the study is stored at the local centres.

Since it should be impossible to identify a specific patient, data are sealed by a code and a patient number replaces personal data. All centres keep a logbook of the number of eligible non-participants, including the reasons for not participating.

Patient data are stored during the study and for fifteen years after the study has ended. If the parents consent, corporeal material is also stored for fifteen years after the study has ended. This material may be used in future for a study with the same research aims.

### Ethical approval

The first of October 2008, this study has been given approval by the medical ethics committee of the ErasmusMC Rotterdam. Thereafter, the local medical ethics committees of eleven participating centres gave their approval. In one centre, ethical approval is still being sought. Once a year, a summary of the trial's progress is send to the medical ethics committee of the ErasmusMC, Rotterdam, the Netherlands. Information is provided on the numbers of subjects included, (serious) adverse events, other problems and amendments. New participating centres are still included. We will continue recruiting patients for our trial until we reached the number of 400 participants. Our goal is to have this amount of patients included by the first of October 2013.

## Discussion

Congenital diaphragmatic hernia (CDH) is associated with a high risk pulmonary morbidity [6]. Several previous studies have reported a wide range of pulmonary problems throughout childhood in these patients [6]. A recent study of our group in collaboration with the CDH Registry reported a 41% prevalence of bronchopulmonary dysplasia (BPD) in survivors of CDH [9]. This study reported the initial ventilation mode to be possibly associated with BPD in these patients. This formed the rationale for our study, which aims at comparing HFO and conventional ventilation as initial ventilation modes in infants with CDH.

Our study is the first randomized clinical trial in the field of CDH. Previous studies were retrospective or observational reports, mostly from single centres. Moreover, progress in CDH research is hampered due to small numbers of patients and lack of evidence-based treatment strategies. Cooperation between centres is therefore highly important to enhance research, exchange knowledge and compare data in larger groups of patients. The CDH Study Group is an example of a worldwide network which has established a large database on CDH patients. To date, the CDH Study Group has published numerous reports in the field of CDH, which contain valuable information on survival, treatment strategies and morbidity in infants with CDH. Another example is the CDH-EURO consortium, a European collaboration between tertiary centres with an expertise in treatment of CDH, which also initiated the VICI-trial. The consortium also developed a recently published standardized treatment protocol [4]. This treatment protocol makes valuable comparison of patient data possible, since all CDH patients, including the VICI-trial patients, born in these centres are treated according to this protocol.

HFO and conventional ventilation are both safe, effective and widely used strategies to ventilate infants with respiratory distress. Although observational and retrospective studies have suggested that HFO may improve survival and pulmonary outcome in infants with CDH, no prospective randomized controlled trials have been carried out to compare initial ventilation strategies in these patients. Therefore, no specific conclusions about certain benefits or disadvantages of either HFO or CMV can be made.

As the first randomized clinical trial in CDH infants, the VICI-trial will provide a clearer view on ventilatory treatment strategies and possible prevention of mortality and morbidity. Still, there are many questions left in the field of CDH and clinical trials are highly needed to improve care and establish standardized treatment. Cooperative networks between centres, such as the CDH-EURO consortium, play a major role in this.

## Competing interests

The authors declare that they have no competing interests.

## Authors' contributions

Contributors: LH wrote the research proposal and the statistical analysis plan, drafted and revised this paper, and currently monitors the data collection of this trial; DT initiated and supervises this trial, drafted and revised the paper, and currently monitors the data collection of this trial; SV currently monitors the data collection of this trial; HB assisted in setting up this trial and currently monitors the data collection of this trial; WH wrote the statistical analysis plan and revised the paper; IR initiated and supervises this trial, drafted and revised the paper, and currently monitors the data collection of this trial. All authors gave final approval of this manuscript to be published.

## Pre-publication history

The pre-publication history for this paper can be accessed here:

http://www.biomedcentral.com/1471-2431/11/98/prepub
